# Publication across Different Languages and Audiences: A Short Personal Note

**DOI:** 10.31662/jmaj.2025-0242

**Published:** 2025-06-27

**Authors:** Shigeki Matsubara

**Affiliations:** 1 Department of Obstetrics and Gynecology, Jichi Medical University, Tochigi, Japan

**Keywords:** duplicate publication, manuscript, paper, retraction, transparency

## Dear Editors,

The latest issue of the *JMA Journal* published a retraction notice ^(1)^: “A third party pointed out that the article was a duplicate publication of a previously published article. Then, the authors contacted the Support Office. The editor-in-chief has therefore decided to retract the article.”

Although the specific prior publication was not named, I suspect it was a Japanese-language review in a formal subspecialty journal. The content overlapped. According to International Committee of Medical Journal Editors guidelines ^(2)^, such overlap―regardless of different language or different target audience―is considered duplicate publication unless disclosed and approved by both editors-in-chief in advance.

From a generalist reader’s perspective, this retracted article was educational. I would not have come across the Japanese version. To me, this case feels somewhat different from a typical instance of intentional misconduct. Nonetheless, current standards must be applied. All parties (i.e., authors, readers, journal, editors, and the editor-in-chief) may have been left unhappy.

I understand the journal may have intentionally kept the notice short and factual. However, hopefully, a brief clarification―such as “duplicate publication includes those in other languages or targeting different audiences”―might be helpful for future authors unfamiliar with these standards.

Complete republication, or simultaneous or joint publication, may sometimes be allowed under specific conditions, including transparency and mutual editorial approval ^(2)^. That path, if followed, might have led to a different outcome in this case. I personally miss that possible scenario.

This is a sensitive matter, and thus I have written this Letter very briefly and flatly. As someone who has long struggled with academic writing, I believe this quiet observation may be of some use to the academic community. I do not criticize anyone involved, and I have no intention to revisit an issue that others may prefer to move on from. I simply hope this short note may help others, especially future authors, navigate similar situations.

## Article Information

### Conflicts of Interest

None

### Author Contributions

Shigeki Matsubara: Manuscript writing.

### Approval by Institutional Review Board (IRB)

Not applicable.

### Patient Anonymity

Not applicable.

### Informed Consent

Not applicable.

### Data Availability

Data sharing is not applicable to this article, as no new data were created or analyzed in this study.
